# High-Fidelity Simulation Scenario: Pediatric Sulfonylurea Overdose and Treatment

**DOI:** 10.15766/mep_2374-8265.10965

**Published:** 2020-09-02

**Authors:** Vincent Calleo, Jacob Anderson, Patrick Curtin, William Paolo

**Affiliations:** 1 Pediatric Emergency Medicine Fellow, Department of Emergency Medicine, SUNY Upstate Medical University; 2 Pediatric Critical Care Fellow, Department of Pediatrics, University of Rochester Medical Center; 3 Medical Student, Department of Education, SUNY Upstate Medical University; 4 Residency Program Director, Department of Emergency Medicine, SUNY Upstate Medical University

**Keywords:** Toxicology, Seizure, Hypoglycemia, Neurology, Competency-Based Medical Education, Competencies, Milestones, EPAs, Interprofessional Education, Pediatric Critical Care Medicine, Pediatric Emergency Medicine, Case-Based Learning, Clinical Teaching/Bedside Teaching, Simulation

## Abstract

**Introduction:**

Oral antidiabetic medications are becoming increasingly popular as the incidence of type II diabetes mellitus increases. Overdoses of these medications, either intentional or accidental, can be detrimental if not quickly recognized and treated. One of the most common classes of hypoglycemic oral antidiabetics, sulfonylureas, was discussed in this case.

**Methods:**

We designed this high-fidelity simulation to help increase the learner's knowledge of sulfonylurea overdoses, including recognizing signs/symptoms, management, and disposition. This simulation was designed to be used with fourth-year medical students, emergency medicine residents, and pediatric residents. The case involves a previously healthy 3-year-old male presenting with altered mental status and seizures secondary to glyburide ingestion. A standard pediatric simulation mannequin was required. The patient presented with altered mental status and began seizing upon arrival. After a thorough history, glyburide ingestion was identified. Critical actions included obtaining a fingerstick glucose measurement, determining an appropriate concentration of dextrose, starting a dextrose drip, and admission for further management.

**Results:**

This simulation case was performed at the simulation lab at SUNY Upstate Medical University by a combination of 83 fourth-year medical students, and emergency medicine and pediatric residents. Feedback and evaluations for the case showed it improved medical education and clinical skills.

**Discussion:**

This simulation was well received and helped participants develop a better understanding of sulfonylurea overdose identification. It also improved participants' ability to manage refractory hypoglycemia and compile a more comprehensive list of differential diagnoses.

## Educational Objectives

By the end of this activity, learners will be able to:

1. Manage an acute seizure in a pediatric patient with an appropriate differential diagnosis.
2. Describe the neurologic, cardiovascular, and systemic effects of acute sulfonylurea toxicity.
3. Manage sulfonylurea overdose including initial and continuous dextrose administration dosing/routes in a pediatric patient.
4. Describe indications for use of octreotide and glucagon.

## Introduction

Diabetes has increased in prevalence over the last several decades. As a result, oral hypoglycemics are being prescribed more frequently. Therefore, the number of accidental pediatric ingestions has increased. Of the oral hypoglycemic agents, sulfonylureas are more likely to require more intense therapy in the case of an overdose, as they are often longer acting than meglitinides. Although overdoses are uncommon, it is necessary for emergency providers to be adept at identifying and treating ingestions when they occur.^1^

This resource was designed to increase awareness and knowledge of oral hypoglycemic medication overdoses. It was designed for fourth-year medical students, emergency medicine residents, and pediatric residents, and was especially ideal for those completing a toxicology rotation. Several papers on oral hypoglycemic have been published, but we did not find any existing simulation cases covering this topic.^2,3^ On *MedEdPORTAL* there were several simulations covering different pathologies of a seizing child, and others teaching pediatric toxicology and ingestion, but there were none describing sulfonylurea ingestion resulting in hypoglycemia and seizures refractory to antiepileptics.^4–7^ This simulation uniquely addresses the treatment and differential diagnoses associated with pediatric seizures and hypoglycemia. We wanted our learners to experience a higher order of cognitive and behavior problem-solving skills, which was provided by a high-fidelity simulation.

This simulation was designed to help assess the learner's awareness of sulfonylurea medication overdose. Its purpose was to help identify gaps in knowledge regarding the stabilization/management of a seizing pediatric patient, initial management of a sulfonylurea overdose, and disposition status post-sulfonylurea ingestion.

## Methods

### Development

We approached the case using a high-fidelity mannequin in a dedicated simulation center. Materials for IV and intraosseus infusion placement were available in the simulation center. A basic understanding of treating seizures, hypoglycemia, and pediatric resuscitation was required in the participants. No prerequisite learning or preparation was required by the participants or facilitators. Here, a pediatric patient presented with seizures after developing an altered mental status. We required learners to stabilize the patient, obtain the appropriate history to diagnose glyburide ingestion, and properly manage and disposition the patient. This, along with checklists, self-assessment tools, and instructor debriefing teaching points, helped familiarize learners with the signs/symptoms of sulfonylurea overdoses and management/disposition.

### Equipment/Environment

The activity occurred in a simulated emergency medicine treatment bay/resuscitation room. The case needed a pediatric mannequin with the capability to produce heart/lung sounds, generate a pulse, and monitor cardiac rhythms. The learners were given various lab values they ordered from the facilitator. Various vials with medication labels were available to help simulate administration in the clinical setting. Available medications included but were not limited to: doses of dextrose in various concentrations (D50, D25, and D10), doses of sodium bicarbonate, succinylcholine, rocuronium, etomidate, fentanyl, versed, normal saline (0.9%), and a liter of D10NS (dextrose 10% in 0.9% sodium chloride).

### Personnel

To successfully complete this simulation, there was a team leader (primary learner). Other participants assisted the team leader and performed actions, such as primary/secondary surveys. However, the team leader made all final decisions.

There was also an instructor controlling the simulation scenario to ensure the activity proceeded as outlined in the critical actions. Two additional facilitators split the roles of bedside nurse, emergency medical services, family members, and toxicologist/poison control center specialist when the appropriate consults were requested

### Implementation

We implemented this simulation during the weekly resident didactic sessions and during toxicology simulation sessions. This simulation was run a total of 10 times during resident didactic days. It was run eight times on the toxicology rotation (consisting of residents and medical students). This simulation was approximately 20 minutes in length with a 10-minute debriefing session. Initially, our first two groups had eight learners which we found to be too large and led to participants being without a role. Therefore, we limited each group to a maximum of five learners. At the start of each simulation, a group leader was selected and team roles were designated by the group leader. The leader was a second- or third-year resident and roles assigned by the leader to participants of all levels of training.

For faculty, there was one person who acted as the preceptor, parent, and consulting physician. One additional person was the simulator operator, responsible for running the mannequin and vital signs. Supplemental materials including the simulation case file (Appendix A), scenario flow sheet (Appendix B), and teaching points (Appendix C) were distributed and reviewed the day before the simulation, and preceptors met 1 hour before to discuss implementation.

### Assessment

There were three different assessment tools used for this simulation. The first tool, a self-evaluation tool (Appendix D), included a Likert scale and was distributed to the learners after completion of the scenario and debriefing session. Learners were asked to identify areas of weakness during the simulation and list key aspects of management to see what was learned during the activity. The second tool, the course evaluation tool (also Appendix D), had the participants numerically rank how successful the simulation was at achieving its learning objectives using a 5-point Likert scale. The final assessment tool, critical actions checklist (Appendix E), had the evaluator track the critical actions and if they were completed. This was filled out by the instructor after completion of the scenario and allowed the outside party to identify areas of weakness that needed to be addressed during the debriefing session. This also helped ensure that all the learning objectives from the simulation were accomplished.

### Debriefing

During the debriefing session, we asked open-ended questions, such as: “How do you think you performed during the simulation?,” “What do you think you did well?,” and, “What do you think you can do to improve in future simulations?” Once these questions were answered, we reviewed the primary and secondary learning objectives of the simulation and highlighted which objectives were and were not met. Once this was completed, we reviewed the etiology, mechanism of action, signs/symptoms, treatment options, and disposition for patients with sulfonylurea ingestions by using the materials covered in Appendix C for teaching points. Finally, we encouraged learners to ask any further questions to help enhance their understanding of this overdose.

## Results

We ran this simulation 18 times with 58 emergency medicine residents, 20 pediatric residents, and five medical students who were going into emergency medicine. All groups were mixed-level learners with at least one second- or third-year resident in each scenario. Four faculty members have used this resource.

All 83 participants (*N* = 83) filled out the postsimulation surveys and the self-assessment tool (Appendix D) after completion of the simulation. Uniformly, the learners stated that they felt more comfortable recognizing, diagnosing, and treating pediatric sulfonylurea overdoses after completing this simulation. One junior resident said the simulation “helped reinforce the importance of checking blood glucose on all critical patients which I often forget.” One senior resident stated the simulation “helped me work through the seizure algorithm and forced me to broaden the differential diagnosis when a case is not going as planned.” Most of the residents also included the importance of considering ingestion when facing persistent hypoglycemia in a pediatric patient. The participants identified that the most common errors in the simulation were failure to check initial blood glucose, to continue a dextrose infusion, and to repeat the blood glucose checks. These were also shown to be the most common missed items in the critical action checklist (Appendix E). This helped drive the focus of the debriefing session.

We used a 5-point Likert scale with 5 meaning strongly agree and 1 meaning strongly disagree. The average score of all our questions for increasing knowledge of sulfonylurea overdoses was 4.5/5. This included the improvement of the participants' comfort in recognizing, treating, and disposition sulfonylurea overdose, which scored an average of 4.3, 4.5, and 4.8, respectively. Participants also rated their improvement of the mechanism of action of sulfonylurea and the antidotes discussed an average score of 4.7. Lastly, the simulation's appropriateness for their level of training was an average of 4.5.

## Discussion

This simulation was designed to help augment awareness of sulfonylurea ingestion and facilitate diagnosis and treatment in an overdose setting. The results of the simulation satisfied the learning objectives. Learners felt more comfortable identifying the signs and symptoms of sulfonylurea overdoses (i.e., recognizing a sulfonylurea overdose). Learners also felt comfortable treating sulfonylurea ingestions after this simulation, and it also increased the learner's knowledge of the topic overall.

This simulation is generalizable, especially given that oral antidiabetic medications are becoming more popular. Sulfonylurea overdoses are likely to be seen in both academic and community practice settings, so understanding how this drug class works in the overdose setting is important to physicians everywhere.

This simulation can be easily incorporated into didactic sessions for both emergency medicine and pediatrics residents as well as pediatric emergency medicine fellows. It can also be run during toxicology rotations for residents and medical students.

There were some limitations we found during the cases. Despite using a high-fidelity infant mannequin there was a lack of urgency that a clinician would have in a real-life situation. In the future, we plan on incorporating a vibration device to simulate the seizing infant. Another limitation was the time constraints due to limited time in the simulation center and limited staff. At our institute, this has been an issue in the past and we plan on incorporating more staff into our simulations to add time for the participants. We did have a 100% response from our 83 participants and our results successfully addressed our learning objectives, but we were limited to student self-assessments (Kirkpatrick's pyramid level 1).^8^ For a more in-depth assessment, we plan on incorporating facilitator evaluations for the participants.

During the pilot phase of this simulation, one challenge was that the dose of octreotide was not included in the case.

Initially, our first two groups were made up of eight participants. We found that the group was too large, and some learners were left without responsibilities. We then limited the groups to four participants which maximized participation and all learners had a responsibility.

One item that helped improve the flow of the simulation was having one faculty member suggest getting a fingerstick glucose 5 minutes into the case if the learner did not ask for one. In some cases, learners anchored on the diagnosis seizure and forgot to check a glucose level. Once redirected, the learners ran through the simulation without significant difficulty. This further led to an emphasis on primary exposure and fingerstick glucose during the debriefing session.

Future opportunities for this simulation include using it for residents on a toxicology rotation, as well as interspersing it throughout the year for general review.

## Appendices

Simulation Case.docxScenario Programming Flow Sheet.docxTeaching Points.docxSelf-Evaluation Tool and Course Assessment Tool.docxCritical Actions Checklist.docx
All appendices are peer reviewed as integral parts of the Original Publication.
