# Supplementary material for: High-Fidelity Simulation Scenario: Pediatric Sulfonylurea Overdose and Treatment
Source: MedEdPORTAL. 2020 Sep 2;16:10965. doi: 10.15766/mep_2374-8265.10965 (PMC7473183; doi:10.15766/mep_2374-8265.10965)
Supplement: Supplementary file 1 — Simulation Case.docxScenario Programming Flow Sheet.docxTeaching Points.docxSelf-Evaluation Tool and Course Assessment Tool.docxCritical Actions Checklist.docx [file mep_2374-8265.10965-s001.zip › A. Simulation Case.docx]

| Appendix A: Simulation Case Template  SIMULATION CASE TITLE: High Fidelity Simulation Scenario: Pediatric Sulfonylurea Overdose and Treatment  AUTHORS: Calleo, MD; Anderson, MD; Curtin, BS; Paolo, MD | |
| --- | --- |
| PATIENT NAME: Johnny  PATIENT AGE: 3 y.o.  CHIEF COMPLAINT: Altered mental status and seizures | |
|  | |
| Brief narrative description of case | Patient is a previously healthy 3-year-old male presenting with altered mental status and seizures secondary to glyburide ingestion. Overall user goals to increase awareness and knowledge of oral hypoglycemic medication overdoses |
| Primary Learning Objectives | Learners should be able to  -Identify symptoms of oral hypoglycemic medication overdose  -Recognize the neurologic, cardiovascular, and systemic effects of acute sulfonylurea toxicity  -Learn appropriate supportive management for this overdose situation, including initial and continuous dextrose administration dosing/routes in a pediatric patient  -Implement fluid resuscitation for cardiovascular effects  -Interpret the metabolic acidosis that can be associated with sulfonylurea medication toxicity. |
| Critical Actions | 1. Ensure that the patient is stable by checking airway, breathing, circulation, disability/dextrose, and ECG/exposure (ABCDE). In addition to the basic ABCDEs, learners should:    1. Administer oxygen via nasal cannula.    2. Obtain a fingerstick glucose measurement    3. Administer a bolus of dextrose, 5cc/kg of D10 or 2cc/kg of D25 (must not be more concentrated than D25 or else line will blow) during seizure activity    4. Obtain and assess initial ECG. 2. Identify sulfonylurea overdose after considering other differential diagnosis.    1. Consult with toxicologist or PCC if needed 3. Initiate management of sulfonylurea overdose.    1. Obtain IV/IO access    2. Administer an ampule of dextrose (not more concentrated than D25) as needed.    3. Start patient on dextrose drip    4. Consider administration of octreotide    5. Consider activated charcoal only if patient is intubated    6. Obtain other laboratory studies (CBC, BMP, VBG, salicylate level, acetaminophen level)    7. Frequently reassess patient, including vital signs and glucose. 4. Disposition - Admit to PICU for further monitoring and treatment 5. Seizure cease and vital signs improve (BP – 90/50 mmHg, HR – 110 bpm) |
| Learner Preparation | N/A |
| Equipment | -Pediatric high-fidelity mannequin  -Various medications including:  -Dextrose (D50, D25, and D10)  -Sodium bicarbonate  -Succinylcholine  -Rocuronium  -Etomidate  -Fentanyl  -Octreotide  -Versed  -Sodium chloride  -D10NS (dextrose 10% in 0.9% sodium chloride) |

| Initial Presentation | | | |
| --- | --- | --- | --- |
| Initial vital signs | T: 37.1°C BP: 74/42 HR: 167bpm RR: 26 breaths/min O_2_ saturation: 85% | | |
| Overall Appearance | Mannequin will be flat but learner will be told patient is having generalized tonic-clonic seizure. | | |
| Actors and roles in the room at case start | -Team leader (primary learner)  -Instructor controlling simulation who may act as PICU attending as well as toxicologist  -Other personnel may play role of EMS and/or patient’s mother/father. | | |
| HPI | Patient brought to ED by EMS after a sudden change in mental status 30 mins prior to arrival. Upon arrival patient begins to have generalized tonic-clonic seizure. The family tells the physician the patient was well until this event began earlier in the day with patient acting “very weird; he seemed really sleepy and wouldn’t respond to much”. Mother initially denies any medications in house aside from aspirin. She does state the patient’s grandmother was babysitting him and she takes glipzide extended release. | | |
| Past Medical/Surgical History | Medications | Allergies | Family History |
| Febrile seizure at 6 months of age | None | None | Uncle with febrile seizures as toddler |
| Physical Examination | | | |
| General | Diaphoretic | | |
| HEENT | Pupils 3mm and nonreactive when seizing; becomes 3mm and reactive when seizing stops | | |
| Neck | Supple, trachea midline | | |
| Lungs | CTAB | | |
| Cardiovascular   \|  \| \| --- \| | Tachycardic.  S1&S2 heard with no murmurs, rubs, or gallops | | |
| Abdomen | Soft, nontender, nondistended | | |
| Neurological | Generalized tonic-clonic seizure, unresponsive to painful stimuli. Once seizures stop, pt’s GCS increases to 15. Pt is still post-ictal. | | |
| Skin | Diaphoretic | | |
| GU | Penis and testis normal | | |
| Psychiatric | Unremarkable | | |

| Instructor Notes - Changes and CASE Branch Points | | |
| --- | --- | --- |
| Intervention / Time point | Change in Case | Additional Information |
| Immediately on arrival | Patient begins to have generalized tonic-clonic seizure |  |
| Participant requests fingerstick glucose measurement | Measured at 27 mg/dL |  |
| Administration of ampule of dextrose (concentration of D25 or less) | Seizure activity stops after administration | Vital signs improve: BP 90/50, HR 110bpm |
| Patient started on dextrose drip |  |  |
| Consult with toxicologist/PCC | Identify sulfonylurea overdose |  |
| Admit to PICU |  |  |

Labs

Hematology Reference

WBC – 11 K/µL (4-10 K/µL)

RBC – 4.7 M/µL (4.1-5.3 M/µL)

Hct – 39 % (36 - 45 %)

Hb – 13.1 g/dL (11.5 – 15.5 g/dL)

Platelets – 375 K/µL (150 – 400 K/µL)

Basic Metabolic Panel Reference

Na – 139 mmol/L (137 – 145 mmol/L)

K – 4.4 mmol/L (3.5 – 5.0 mmol/L)

Cl – 100 mmolL (98 – 107 mmol/L)

CO2 – 26 mmol/L (22 – 30 mmol/L)

Anion Gap – 13 mmol/L (3 – 13 mmol/L)

Glucose – 27 mg/dL (70 – 105 mg/dL)

BUN – 12 mg/dL (7 – 17 mg/dL)

Creatinine – 1.1 mg/dL (0.7 – 1.2 mg/dL)

Ca – 9.3 mg/dL (8.4 – 10.3 mg/dL)

Mg – 1.6 meq/L (1.3 – 1.9 meq/L)

Phosphorus – 4.1 mg/dL (2.5 – 4.5 mg/dL)

Blood Gas Reference

pH – 7.29 (7.38 – 7.44)

pCO_2_ – 29 (35 – 40 mmHg)

pO_2_ – 89 (95 – 100 mmHg)

O_2_ saturation – 93 % (94 – 100 %)

Ethanol Level Reference

Ethanol – 0.00 gm/dL (Negative)

Additional Tests Reference

Salicylate – Negative Negative

Acetaminophen – Negative Negative

EKG

Sinus tachycardia. PR=.155s, QRS=.78s, QTc=425ms

Ideal Scenario Flow

The learner enters the room to find the patient seizing. The learner immediately checks patient stability by checking breathing, circulation, disability/dextrose, and ECG/exposure. A fingerstick glucose measurement, ECG, other laboratory studies (CBC, BMP, VBG, salicylate level, acetaminophen level) are obtained. Oxygen is administered by nasal cannula. The learner gives the patient an initial ampule of dextrose (D25 or less) after obtaining IV/IO access. The patient is started on a dextrose drip and the learner considers administration of octreotide and activated charcoal if the patient is intubated. The learner consults the toxicologist and sulfonylurea overdose is identified after considering the differential diagnosis. The learner admits the patient to the PICU for further monitoring and treatment.

Anticipated Management Mistakes

1. *Unable to identify hypoglycemia as cause of seizure:* Students often became fixated with treating the seizure with benzodiazepines rather than identifying and treating the underlying cause. We found it useful to have the nurse prompt with, “Should we check anything in the meantime?” five minutes after the case begins to help put students back on the right track.
2. *Students continue to use D50:* Some learners continued to try to use multiple boluses of D50 to treat hypoglycemia. We found it useful to continue telling them the line blew to prompt them to think of using a different concentration. After the second time of incorrectly dextrose, the nurse may prompt with, “Should we try something different?”

Debriefing Plan:

1. The simulation participant, on completion, should go over the details of the case with the simulation instructors and all relevant sulfonylurea overdose signs, symptoms, and treatments as outlined in the critical actions. Each treatment option should be outlined and discussed. Instructors should be sure to ask the participant to explain why they did or did not administer octreotide, glucagon, and activated charcoal; once the participant answers, the logic behind each decision should be discussed.
2. The importance of looking for reversible causes of seizures in a pediatric patient should be reinforced. Instructors should also emphasize the importance of obtaining a comprehensive medication history from family members of pediatric patients. Learners should also be reminded of the importance of giving proper pediatric concentrations of dextrose.
3. A brief review of the importance, mechanism of action, signs/symptoms, treatment, and disposition of sulfonylurea overdoses should be discussed, as outlined in the Teaching Points (appendix C).
