# Supplementary material for: High-Fidelity Simulation Scenario: Pediatric Sulfonylurea Overdose and Treatment
Source: MedEdPORTAL. 2020 Sep 2;16:10965. doi: 10.15766/mep_2374-8265.10965 (PMC7473183; doi:10.15766/mep_2374-8265.10965)
Supplement: Supplementary file 1 — Simulation Case.docxScenario Programming Flow Sheet.docxTeaching Points.docxSelf-Evaluation Tool and Course Assessment Tool.docxCritical Actions Checklist.docx [file mep_2374-8265.10965-s001.zip › B. Scenario Programming Flow Sheet.docx]

**Appendix B:** Scenario Programming Flow Sheet

- Please see Critical Actions Checklist in Appendix E


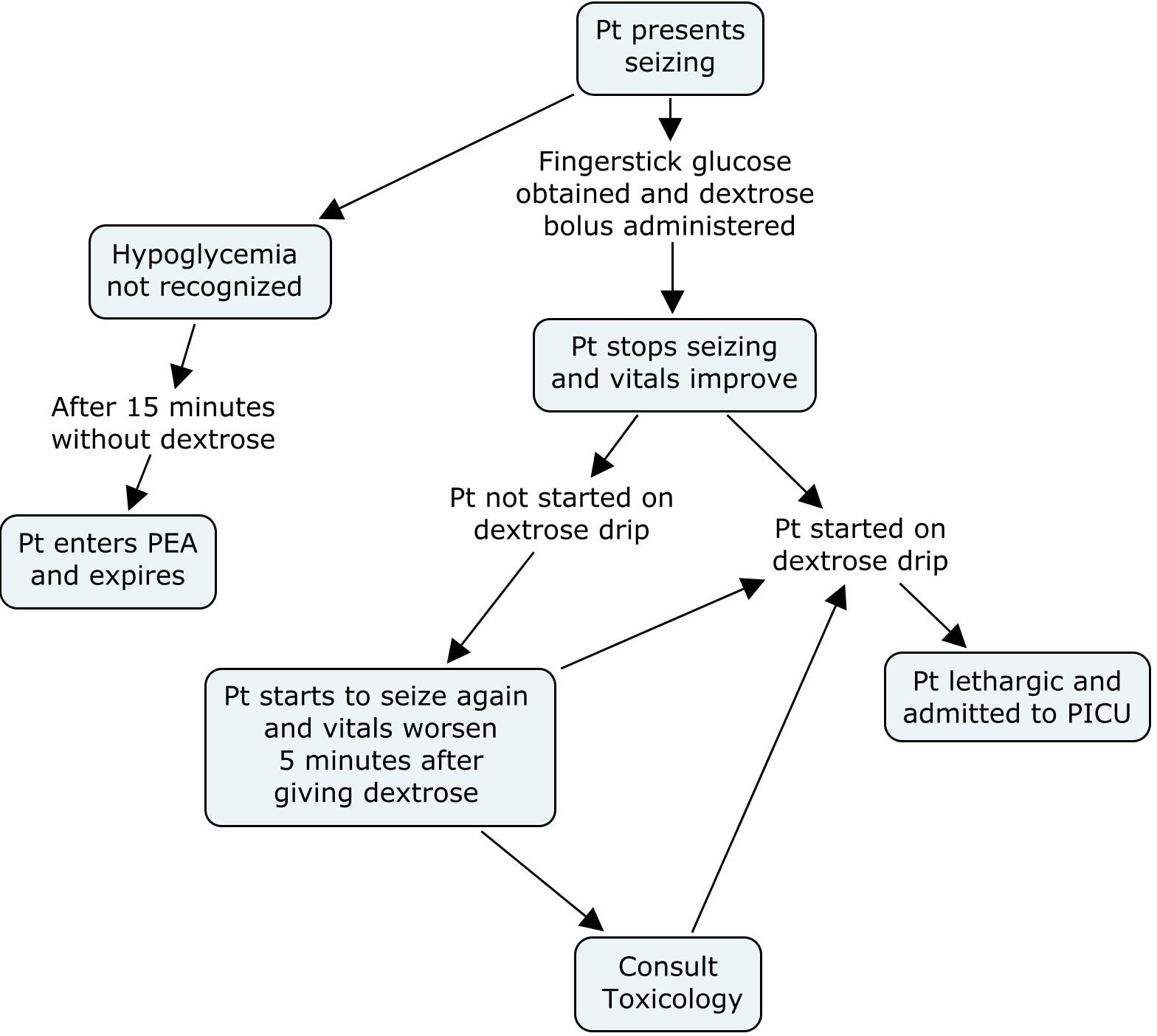


*Author created image
