# Supplementary material for: High-Fidelity Simulation Scenario: Pediatric Sulfonylurea Overdose and Treatment
Source: MedEdPORTAL. 2020 Sep 2;16:10965. doi: 10.15766/mep_2374-8265.10965 (PMC7473183; doi:10.15766/mep_2374-8265.10965)
Supplement: Supplementary file 1 — Simulation Case.docxScenario Programming Flow Sheet.docxTeaching Points.docxSelf-Evaluation Tool and Course Assessment Tool.docxCritical Actions Checklist.docx [file mep_2374-8265.10965-s001.zip › C. Teaching Points.docx]

**Appendix C:** Teaching Points

*Importance*:

The diabetes epidemic has worsened over the last several decades; some estimates predict that by 2025, over 300 million individuals will have diabetes mellitus (DM)^1^. Oral hypoglycemic medication use will likely increase to help treat this problem, and as such, accidental ingestions may increase. Of the oral hypoglycemic medications, two of the most common types are sulfonylureas and meglitinides. Sulfonylureas are more likely to require more intense therapy, as they are often longer acting than meglitinides.

*Mechanism of Action*:

Sulfonylureas bind ATP-sensitive potassium channels. This results in beta-islet cell hyperpolarization by inhibiting potassium efflux^2^. This leads to an opening of calcium channels that then leads to insulin release from secretory granules.

Octreotide inhibits voltage gated calcium channels, which blocks calcium efflux and impedes the release of insulin from beta-islet cells.

*Signs/Symptoms:*

Hypoglycemia is the most commonly seen complication, as most oral hypoglycemic work increasing insulin release. After patients become hypoglycemic, symptoms include lightheadedness, nausea, and confusion. Tachycardia and hypotension sometimes arise. If hypoglycemia is profound, diaphoresis and seizures can also occur. If hypoglycemia is continually untreated, hemiparesis, neurologic deficits, and metabolic acidosis are occasionally seen^3^.

*Interventions*:

For most sulfonylurea overdoses, supportive management is all that is required. Dextrose boluses are first-line therapy if patient is unable to tolerate PO glucose^4^. If the patient’s glucose is refractory to the dextrose bolus, a continuous infusion should be started. A good way to remember the dosing of a dextrose bolus is the "rule of 50." In the event that this doesn’t provide an adequate response, the patient should be started on octreotide^5,6^. Glucagon isn’t routinely recommended^7^; one case it may be considered is when IV access cannot be obtained, as patients with sulfonylurea overdoses rapidly deplete their glucagon stores. Activated charcoal is recommended for sulfonylurea overdoses if the ingestion occurred within the last hour; it is not routinely recommended if the ingestion time is unknown. Activated charcoal should not be administered if the patient cannot protect his/her own airway.

*Disposition*:

Disposition with a pediatric overdose can be tricky and is often clinician dependent. The pharmacokinetics of sulfonylureas can vary. In general, most sulfonylurea ingestions require a 24-hour monitoring period if the patient is asymptomatic. If the patient develops symptoms, he/she should be served for a minimum of 24 hours or until the patient is asymptomatic (whichever comes later).

**References**

1. King H, Aubert RE, Herman WH. Global burden of diabetes, 1995-2025: Prevalence, numerical estimates, and projections. *Diabetes Care*. 1998;21(9):1414-1431.

2. Harrigan RA, Nathan MS, Beattie P. Oral agents for the treatment of type 2 diabetes mellitus: Pharmacology, toxicity, and treatment. *Ann Emerg Med*. 2001;38(1):68-78.

3. Spiller M, ABAT HA, Schroeder M, Sharon L, Ching M, Donna S. Hemiparesis and altered mental status in a child after glyburide ingestion. *J Emerg Med*. 1998;16(3):433-435.

4. Spiller HA. Management of antidiabetic medications in overdose. *Drug Safety*. 1998;19(5):411-424.

5. Dougherty PP, Klein-Schwartz W. Octreotide’s role in the management of sulfonylurea-induced hypoglycemia. *Journal of Medical Toxicology*. 2010;6(2):199-206.

6. Green RS, Palatnick W. Effectiveness of octreotide in a case of refractory sulfonylurea-induced hypoglycemia. *J Emerg Med*. 2003;25(3):283-287.

7. Spiller HA, Sawyer TS. Toxicology of oral antidiabetic medications. *Am J Health Syst Pharm*. 2006;63(10):929-938.

8. Little GL, Boniface KS. Are one or two dangerous? sulfonylurea exposure in toddlers. *J Emerg Med*. 2005;28(3):305-310.
