# Supplementary material for: High-Fidelity Simulation Scenario: Pediatric Sulfonylurea Overdose and Treatment
Source: MedEdPORTAL. 2020 Sep 2;16:10965. doi: 10.15766/mep_2374-8265.10965 (PMC7473183; doi:10.15766/mep_2374-8265.10965)
Supplement: Supplementary file 1 — Simulation Case.docxScenario Programming Flow Sheet.docxTeaching Points.docxSelf-Evaluation Tool and Course Assessment Tool.docxCritical Actions Checklist.docx [file mep_2374-8265.10965-s001.zip › D. Self-Evaluation Tool and Course Assessment Tool.docx]

**Appendix D**: Self-Evaluation Tool

- To be submitted after completion of simulation

List three items that you learned from this simulation that you did not know prior to participation in the exercise:

1. ______________________________________________________________________
2. ______________________________________________________________________
3. ______________________________________________________________________

If an error occurred during your simulation, what did you learn from it and how will you avoid making this in the future?

Discuss the signs/symptoms that can be seen in patients with a sulfonylurea overdose:

Describe the various treatment options for a sulfonylurea overdose and when each treatment option is indicated:

**Appendix D:** Course Assessment Tool

- To be submitted after completion of simulation

Please rate the following statements from 1 (strongly disagree) to 5 (strongly agree)

I am comfortable recognizing a pediatric patient with a sulfonylurea overdose

1 2 3 4 5

I am comfortable treating a pediatric patient with a sulfonylurea overdose

1 2 3 4 5

This simulation increased my awareness of sulfonylurea medication overdose

1 2 3 4 5

The simulation improved my knowledge of the mechanism of action of sulfonylureas

1 2 3 4 5

The simulation enhanced my awareness of when to use various treatment modalities in treating a sulfonylurea overdose scenario

1 2 3 4 5

The simulation helped improve my confidence in dispositioning a patient with a sulfonylurea overdose

1 2 3 4 5

This simulation was appropriate for my level of training

1 2 3 4 5

Additional Comments:
