# Supplementary material for: High-Fidelity Simulation Scenario: Pediatric Sulfonylurea Overdose and Treatment
Source: MedEdPORTAL. 2020 Sep 2;16:10965. doi: 10.15766/mep_2374-8265.10965 (PMC7473183; doi:10.15766/mep_2374-8265.10965)
Supplement: Supplementary file 1 — Simulation Case.docxScenario Programming Flow Sheet.docxTeaching Points.docxSelf-Evaluation Tool and Course Assessment Tool.docxCritical Actions Checklist.docx [file mep_2374-8265.10965-s001.zip › E. Critical Actions Checklist.docx]

**Appendix E: Critical Actions Checklist**

**Case: Sulfonylurea Overdose and Treatment**

**Date:______________ Time: _____________**

Not Performed with Performed

**Critical Actions Checklist** Performed Prompting/Help Well

1. **Recognize and treat hypoglycemia** **⁯ ⁯ ⁯**
   1. Obtained initial fingerstick glucose **⁯ ⁯ ⁯**
   2. Obtained IV/IO access **⁯ ⁯ ⁯**
   3. Administer appropriate amp of dextrose **⁯ ⁯ ⁯**
   4. Frequently recheck glucose **⁯ ⁯ ⁯**
   5. Repeat amp of dextrose if hypoglycemic **⁯ ⁯ ⁯**
   6. Initiate dextrose drip **⁯ ⁯ ⁯**
2. **Identify sulfonylurea overdose**  **⁯ ⁯ ⁯**
   1. Obtain appropriate history from family **⁯ ⁯ ⁯**
   2. Consult with toxicology **⁯ ⁯ ⁯**
3. **Manage sulfonylurea overdose**  **⁯ ⁯ ⁯**
   1. Amp of dextrose administered **⁯ ⁯ ⁯**
   2. Dextrose infusion started **⁯ ⁯ ⁯**
   3. Consider administration of octreotide **⁯ ⁯ ⁯**
   4. Administer appropriate fluid bolus **⁯ ⁯ ⁯**
   5. Do not administer charcoal unless **⁯ ⁯ ⁯**

patient is intubated d/t mental status change

- 1. Reassess patient, including vitals **⁯ ⁯ ⁯**

and repeat glucose measurements
